# Clinical characterization, natural history, and neuroimaging of cerebellar ataxia after abdominal surgery

**DOI:** 10.1055/s-0045-1809934

**Published:** 2025-07-15

**Authors:** Victor Rebelo Procaci, Thiago Yoshinaga Tonholo Silva, Raphael Pinheiro Camurugy da Hora, Orlando Graziani Povoas Barsottini, José Luiz Pedroso

**Affiliations:** 1 Universidade Federal de São Paulo, Escola Paulista de Medicina, Departamento de Neurologia e Neurocirurgia, Unidade de Ataxias, São Paulo SP, Brazil.

**Keywords:** Postoperative Complications, Ataxia, Bariatric Surgery, Avitaminosis

## Abstract

**Background:**

The rates of complications after major abdominal surgeries remain high, despite the advances in pre- and postoperative care and surgical techniques. In these cases, neurological disorders mainly include stroke and delirium, with a high increase of morbidity. Ataxia is rarely a consequence of abdominal procedures like, more often being related to long-term complications of bariatric surgery due to chronic vitamin deficiency.

**Objective:**

To describe seven cases of ataxia following major abdominal surgeries and propose a prophylactic approach.

**Methods:**

A retrospective case series in which medical records of patients from the Ataxia Unit of Universidade Federal de São Paulo were evaluated from January 2007 to August 2024. We identified seven patients who developed acute cerebellar ataxia after gastrointestinal surgery. Demographic, clinical, laboratory, neuroimaging, and treatment data were extracted. Descriptive statistics was used to summarize findings.

**Results:**

There were two cases that evolved with neurological improvement, and five remained with severe cerebellar ataxia. Brain imaging showed cerebellar atrophy in three patients and signs of Wernicke encephalopathy in two.

**Conclusion:**

This case series describes an unusual form of acute ataxia with poor outcomes, possibly related to complications from major abdominal surgery. Early intervention and prophylactic supplementation with vitamins B1 and B12 in patients receiving TPN should be considered to avoid such severe neurological complications.

## INTRODUCTION

The term *ataxia* , from the Greek word *taxis* , meaning *order* , denotes a disorder of coordination and balance. 1 2 This condition comprises a wide spectrum of neurological disorders and may be caused by disturbances in several parts of the nervous system (such as the cerebellum, brainstem, spinal cord, and peripheral nerves). Acute ataxias can be caused by several medical conditions, ranging from infectious, vascular, inflammatory, toxic, and metabolic, among others. 3 It may manifest in some patients after abdominal surgery as a rare complication, which could have different etiologies. 4 5 6 7

Postoperative complication rates for major abdominal surgeries remain high, despite the advances in preoperative and postoperative care and surgical techniques. 8 Elective procedures have complications in up to 50% of the patients, while emergency abdominal surgeries have a high complication rate that can reach 70%. 9

Neurological disorders after large abdominal procedures mainly include stroke and delirium, with a high increase of morbidity. 8 Other neurological complications of abdominal procedures, such as ataxia, are rare, and are more often long-term complications of bariatric surgery due to chronic vitamin deficiency. 5

The aim of this case series is to increase the recognition of cerebellar ataxia as a serious, and eventually irreversible, neurologic complication caused by gastrointestinal surgery. As well as to discuss the possible pathophysiological mechanisms related to postsurgical ataxia caused by vitamin deficiency.

## METHODS

The present is a retrospective, observational, and descriptive case series based on the review of medical records from the Ataxia Unit of Universidade Federal de São Paulo, between January 2007 and September 2024. We evaluated our database with 1,410 patients with different forms of ataxia (hereditary, acquired, and degenerative) and identified seven patients with a diagnosis of acute cerebellar ataxia that developed after major abdominal surgery. We collected demographic data (age, sex), surgical details (type of surgery, indication, complications), nutritional information (use and duration of total parenteral nutrition [TPN]), comorbidities, medications used during hospitalization, neurological findings, laboratory results, neuroimaging findings, treatments administered, and clinical outcomes. All data were analyzed descriptively.

The study was approved by our Ethics Committee under the number 82532224.8.0000.5505, and informed consent for participation in this report was obtained from all patients.

## RESULTS

All seven patients developed acute-onset ataxia during postoperative hospitalization following abdominal surgery. Clinical and demographic data were analyzed, including sex (4 female and 3 male subjects), age at symptom onset (range: 27–54 years), type of surgery and complications, medications administered during hospitalization, medical history, duration of TPN, neurological findings, laboratory tests relevant to ataxia evaluation, neuroimaging features, treatment, suspected etiology of ataxia, and clinical outcomes.

All seven patients underwent a major abdominal surgery. The initial surgical diagnosis was variable: two were elective (bariatric surgery) and five were emergency procedures (blunt abdominal trauma with intestinal perforation, penetrating abdominal trauma, acute appendicitis, and intestinal occlusion).

Furthermore, five of the patients had to perform at least one more procedure due to complications: infection (peritonitis, and surgical wound infection) and occlusion (intestinal obstruction).

During the hospital stay, all seven patients received TPN for a period that ranged from 7 days to 3 months. Detailed nutritional information is not available. Four patients received multiple antibiotics, and for the other three the information is absent from the medical records. Obesity (2/7), diabetes mellitus and hepatitis C virus infection (1/7), hypertension (1/7), and tobacco and alcohol consumption (1/7) were reported previous issues. None had previous history of neurologic disorder.

Following the procedure, the seven patients have presented acute/subacute onset of global cerebellar ataxia, characterized by limb dysmetria, dysdiadochokinesia, and gait ataxia. Other main features include nystagmus in six of the patients, and loss of deep sensation modalities (vibration and proprioception) in four of them.

**Figure 1:**
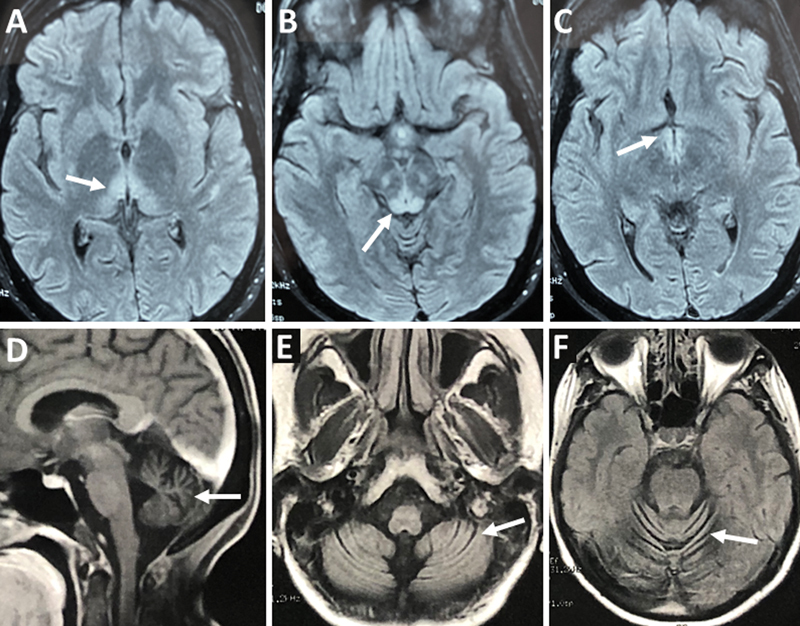
Patient 1: axial fluid-attenuated inversion recovery (FLAIR)-weighted brain magnetic resonance imaging (MRI) scan showing bilateral hyperintense signal in the medial thalami ( **A** ), periaqueductal grey matter ( **B** ), and mammillary bodies ( **C** ), characterizing the typical imaging findings of Wernicke encephalopathy. Patient 3: sagittal T1-weighted ( **D** ) and axial FLAIR-weighted ( **E, F** ) brain MRI discloses cerebellar atrophy.

Brain magnetic resonance imaging (MRI) scans disclosed T2 symmetrically increased intensity in the periaqueductal grey matter of two patients, who were diagnosed as Wernicke encephalopathy, and cerebellar atrophy in three patients ( Figure 1 ). The remaining two patients had no brain MRI abnormalities. Vitamin supplementation (thiamine ± cobalamin) was prescribed to six patients; the other one did not have information about vitamin use. However, only two patients had improvement in gait and limb coordination.

In 2020, Patient 4 presented to our clinic with dysarthria, gaze-evoked nystagmus, dysmetria, and dysdiadochokinesia of the limbs, truncal ataxia with constant sway, and gait ataxia with a wide base, being unable to perform tandem walking. After 11 months of vitamin supplementation with oral thiamine (300 mg/day) and monthly intramuscular cobalamin (5,000 mcg), he exhibited complete resolution of dysarthria and gait ataxia, and could walk in tandem. Gaze-evoked nystagmus and mild upper limb dysmetria persisted.

Patient 6, evaluated in 2020, presented with gaze-evoked nystagmus, dysmetria, and dysdiadochokinesia of the limbs, broad-based gait with intermittent support, along with other neurological findings including reduced deep sensation below the knees, patellar and Achilles areflexia, and mild lower limb weakness (Medical Research Council grade 4). After 13 months of supplementation with oral thiamine (300 mg/day), oral folate (5 mg/day), and monthly intramuscular cobalamin (5,000 mcg), she showed progressive neurological improvement. By 2021, nystagmus and dysdiadochokinesia were absent, and she was able to walk unaided with a narrow-based gait. Muscle strength recovered completely, while tendon reflexes demonstrated partial improvement.

The other five patients maintained chronic, nonprogressive limb and gait ataxia. Table 1 summarizes clinical features, biomarkers, and neuroimaging of all seven patients evaluated. A **Supplementary Material** (available at https://www.arquivosdeneuropsiquiatria.org/wp-content/uploads/2025/05/ANP-2025.0059-Supplementary-Material.docx ) containing the laboratory workup of each patient is provided in a separate document.

**Table 1:** Clinical profile of postgastrointestinal surgery patients who developed cerebellar ataxia

	Patient 1	Patient 2	Patient 3	Patient 4	Patient 5	Patient 6	Patient 7
Age at onset (years)	35	54	31	43	34	40	27
Sex	Male	Female	Female	Male	Female	Female	Male
Surgical diagnosis; year	Acute appendicitis; peritonitis; 2019	Obesity surgery; intestinal obstruction; 2018	Obesity surgery; intestinal obstruction; 2011	Penetrating abdominal trauma; 2018	Acute appendicitis; peritonitis; perforated gastric ulcer; 2018	Total colectomy; surgical wound infection; 2020	Blunt trauma, intestinal perforation, sepsis, colectomy and colostomy; colostomy reversed after 2 years; 2007
TPN duration	3 weeks	10 days	> 1 week	4 weeks	3 weeks	4 weeks	3 months
Ataxia onset after surgery	4 weeks	Unknown	1 week	7 weeks	6 weeks	4 weeks	After surgery, noticed at hospital discharge (> 3 months)
Ataxia	Axial, appendicular	Axial, appendicular	Axial, appendicular	Axial, mild appendicular	Axial, appendicular	Axial, mild appendicular	Axial, appendicular
Year of last consultation	2020	2020	2021	2022	2022	2021	2024
Other neurological features	None	Horizontal nystagmus	Nystagmus, cervical and upper limbs dystonic tremor	Gaze-evoked nystagmus, peripheral neuropathy, dysarthria	Downbeat nystagmus, peripheral neuropathy, dysarthria	Gaze-evoked nystagmus, peripheral neuropathy	Gaze-evoked nystagmus, peripheral neuropathy, dysarthria
Medical history	None	Obesity	Obesity	Alcohol and tobacco use	None	Diabetes, treated HCV	Hypertension, unilateral nephrectomy, nephrolithiasis
Brain MRI; year	T2 hyperintense medial thalami, periaqueductal, and mammillary bodies; 2020	Normal; 2020	Cerebellar atrophy; 2019	Mild cerebellar atrophy; 2020	Normal; 2018 and 2019	T2 hyperintense medial thalami and periaqueductal grey matter; 2020	Mild cerebellar atrophy; 2024
Treatment	B1	B1, B12	B1, B12, folate	B1, B12	B1, B12	B1, B12, folate	Unknown – thiamine and cobalamin started 17 years after symptom onset, when referred to our center
Improvement	None	None	None	Gait and limb coordination improvement	None	Gait and limb coordination improvement	None

Abbreviations: B1, vitamin B1; B12, vitamin B12; HCV, hepatitis C virus; MRI, magnetic resonance imaging; TPN, total parental nutrition.

## DISCUSSION

Acute-onset, nonprogressive ataxia represents a heterogeneous group of hereditary and sporadic conditions that remain a diagnostic challenge. Thus, frequently, its specific etiology cannot be attained despite extensive investigation. 10

Acute ataxia and other neurological complications after major abdominal surgeries are rare, and more often reported in the setting of bariatric surgery. Most neurological complications in these patients are related to chronic malabsorption syndrome, low nutritional intake, and hyperemesis, leading to micronutrient deficiency. 5 6 Remarkably, malabsorption syndrome and low nutritional intake that cause vitamin deficiency usually lead to tardive complications beginning months after the procedure. 5 However, prior subclinical nutrient deficiency can manifest earlier. 7

### Thiamine deficiency

Cerebellar ataxia can occur in the context of Wernicke encephalopathy due to the critical role of thiamine in carbohydrate metabolism and neurotransmitter function. Thiamine acts as a cofactor for enzymes such as pyruvate dehydrogenase and alpha-ketoglutarate dehydrogenase, which are essential for energy production in neurons. Its deficiency can lead to reversible mitochondrial damage and impaired neurotransmitter uptake, particularly serotonin in the cerebellum, which could contribute to cerebellar dysfunction and ataxia. 11 12

Thiamine deficiency can arise from small intestine resection, low food intake after abdominal surgery, and secondary to persistent nausea and vomiting. It is important to recognize patients at risk, because this is a reversible cause of neurologic disfunction. 4

### Vitamin B12 deficiency

Vitamin B12 deficiency is known to cause several neurological manifestations, including cerebellar ataxia. It can also lead to demyelination in various parts of the nervous system, including the spinal cord, cranial and peripheral nerves, and brain white matter, which can involve the cerebellum. The pathophysiology involves impaired myelination and neuronal function due to the role of vitamin B12 in DNA synthesis and methylation processes. 13 14

Impaired absorption of vitamin B12, can occur after surgical resection of the ileum, bariatric surgeries, and gastrectomy, which leads to deficiency and subsequent neurological dysfunction. Early recognition and treatment with parenteral vitamin B12 can prevent or reverse these neurological deficits, although the degree of recovery depends on duration and severity. 15

### Vitamin E deficiency

Vitamin E deficiency secondary to malnutrition and intestinal surgeries has been reported in the literature, although its clinical manifestations following surgery are rarely documented. 16 17 Malabsorptive procedures, such as bariatric surgeries, particularly Roux-en-Y gastric bypass and biliopancreatic diversion, are associated with an increased risk of vitamin E deficiency due to impaired absorption of fat-soluble vitamins. Additionally, gastrectomy, whether partial or total, can lead to this deficiency. The optimal dose of supplementation required for prevention of deficiency or for treatment following bariatric surgery has yet to be established. 16

### Copper deficiency

Copper deficiency can result from certain types of abdominal surgery, such as gastric bypass or gastrectomy. This condition is characterized by a clinical presentation similar to the subacute combined degeneration seen in vitamin B12 deficiency, with symptoms including sensory ataxia, myelopathy, and peripheral neuropathy. Cerebellar atrophy in copper deficiency has been reported, but it is unclear if this area is directly affected. 18 19

### Metronidazol toxicity

Metronidazole, an antibiotic commonly used in gastrointestinal infections and in the context of abdominal surgeries, can cause cerebellar ataxia due to neurotoxicity. This is characterized by MRI findings of high signal intensity in the dentate nuclei and is typically reversible upon discontinuation of the drug. Long-term or high-dose use of metronidazole increases the risk of such adverse effects. 20 21 22

### Other mechanisms

A study conducted in 2019 23 recruited 465 children with acute cerebellar ataxia. Of them, 261 had performed intestinal surgery. From the surgery group, 30 patients were randomly assigned to collect stool samples for microbiome analysis; they were compared with 12 patients with no intestinal surgery and 10 healthy controls. The authors found altered genera and phyla associated with acute cerebellar ataxia and proposed a possible association between changes in gut microbiome after intestinal surgery via the gut-brain axis. However, the authors do not specify if vitamin deficiencies were present.

Our seven subjects compose a heterogeneous population, predominantly female, with different risk factors to neurologic manifestations. Besides the complicated gastrointestinal procedures, the main common point among the patients was the necessity of postoperative TPN. In a high catabolic state, it might not sufficiently meet the increased necessity of micronutrients, raising the risk of nutritional deficiency.

The female preponderance of our sample reinforces the nutritional etiology, as women are more prone to developing thiamine deficiency syndrome. 7 The toxicity of metronidazole and other drugs might also be an important factor to take on account, as they are commonly used after abdominal procedures. On the other hand, none of our patients had the typical metronidazole-induced encephalopathy with dentate nuclei and/or callosal lesions on MRI. 20 22

It is important to note that our study has some limitations. The retrospective case series design does not allow for the establishment of causality, and all data were obtained through medical record review, depending on the completeness of documentation. Serum biomarkers, vitamin levels, and lists of medications were not available during the period of TPN and soon after surgery complication. Additionally, we did not have access to the medical records from the hospitalization period during which TPN was administered. All seven patients were evaluated several weeks or years after their abdominal procedures.

In conclusion, this case series shows an unusual form of acute ataxia with poor outcomes, possibly related to complications after major abdominal surgery. Also, two patients presented with brain MRI abnormalities compatible with Wernicke encephalopathy, which suggest that vitamin deficiency could be the main underlying cause. New mechanisms involving the gut-brain axis still need to be more investigated.

After a thorough review of our cases and the available literature, we suggest that early supplementation with vitamin B1 (300 mg/day) and B12 (5,000 mcg/week for the first 4 weeks, followed by 5,000 mcg/month) should be considered in patients undergoing major abdominal surgery, even when TPN is used, in order to prevent severe neurological complications, such as ataxia. The route of vitamin administration may vary depending on the clinical case and the type of abdominal surgery, and withdrawal of vitamin supplementation should be considered once the patient can meet their nutritional needs.

## References

[1] Garcin R The ataxias Amsterdam North-Holland 1969 V. 1 309 352

[2] Bonilha P AAM Cassarotti B Nunes T EM Teive H AG Frontal ataxia: historical aspects and clinical definition Arq Neuro-Psiquiatr 2023 81 10 934 936 10.1055/s-0043-1775886 PMC10631853 37899045

[3] Pedroso J L Vale T C Braga-Neto P Dutra L A França M C Jr Teive H AG Barsottini O GP Acute cerebellar ataxia: differential diagnosis and clinical approach Arq Neuro-Psiquiatr 2019 77 03 184 193 10.1590/0004-282X20190020 30970132

[4] Oudman E Wijnia J W Van Dam M Biter L U Postma A Preventing Wernicke Encephalopathy After Bariatric Surgery Obes Surg 2018 28 07 2060 2068 10.1007/s11695-018-3262-4 29693218 PMC6018594

[5] Berger J R Singhal D The neurologic complications of bariatric surgery Handb Clin Neurol 2014 120 587 594 10.1016/B978-0-7020-4087-0.00039-5 24365339

[6] Moss H E Bariatric Surgery and the Neuro-Ophthalmologist J Neuroophthalmol 2016 36 01 78 84 10.1097/WNO.0000000000000332 26764529 PMC4755831

[7] Stroh C Meyer F Manger T Beriberi, a severe complication after metabolic surgery - review of the literature Obes Facts 2014 7 04 246 252 10.1159/000366012 25095897 PMC5644786

[8] Veličković J Feng C Palibrk I Veličković D Jovanović B Bumbaširević V The Assessment of Complications After Major Abdominal Surgery: A Comparison of Two Scales J Surg Res 2020 247 397 405 10.1016/j.jss.2019.10.003 31676144

[9] Tengberg L T Cihoric M Foss N B Bay-Nielsen M Gögenur I Henriksen R Complications after emergency laparotomy beyond the immediate postoperative period - a retrospective, observational cohort study of 1139 patients Anaesthesia 2017 72 03 309 316 10.1111/anae.13721 27809332

[10] Pinto WBVdR Pedroso J L Souza PVSd Albuquerque MVCd Barsottini O GP Non-progressive cerebellar ataxia and previous undetermined acute cerebellar injury: a mysterious clinical condition Arq Neuro-Psiquiatr 2015 73 10 823 827 10.1590/0004-282X20150119 26291991

[11] Mulholland P J Susceptibility of the cerebellum to thiamine deficiency Cerebellum 2006 5 01 55 63 10.1080/14734220600551707 16527765

[12] Plaitakis A Nicklas W J Berl S Thiamine deficiency: selective impairment of the cerebellar serotonergic system Neurology 1978 28 07 691 698 10.1212/wnl.28.7.691 27736

[13] Chakrabarty B Dubey R Gulati S Yoganathan S Kumar A Kumar A Isolated cerebellar involvement in vitamin B12 deficiency: a case report J Child Neurol 2014 29 11 NP161 NP163 10.1177/0883073813513498 24346315

[14] Morita S Miwa H Kihira T Kondo T Cerebellar ataxia and leukoencephalopathy associated with cobalamin deficiency J Neurol Sci 2003 216 01 183 184 10.1016/s0022-510x(03)00219-3 14607321

[15] Guéant J L Guéant-Rodriguez R M Alpers D H Vitamin B12 absorption and malabsorption Vitam Horm 2022 119 241 274 10.1016/bs.vh.2022.01.016 35337622

[16] Sherf-Dagan S Buch A Ben-Porat T Sakran N Sinai T Vitamin E status among bariatric surgery patients: a systematic review Surg Obes Relat Dis 2021 17 04 816 830 10.1016/j.soard.2020.10.029 33323330

[17] Ueda N Suzuki Y Rino Y Takahashi T Imada T Takanashi Y Kuroiwa Y Correlation between neurological dysfunction with vitamin E deficiency and gastrectomy J Neurol Sci 2009 287(1-2):216 220 10.1016/j.jns.2009.07.020 19709675

[18] Moon N Aryan M Westerveld D Nathoo S Glover S Kamel A Y Clinical Manifestations of Copper Deficiency: A Case Report and Review of the Literature Nutr Clin Pract 2021 36 05 1080 1085 10.1002/ncp.10582 33037701

[19] Cao J Ran L Liu C Li Z Serum copper decrease and cerebellar atrophy in patients with nitrous oxide-induced subacute combined degeneration: two cases report BMC Neurol 2021 21 01 471 10.1186/s12883-021-02496-y 34863097 PMC8643018

[20] Patel L Batchala P Almardawi R Morales R Raghavan P Acute metronidazole-induced neurotoxicity: an update on MRI findings Clin Radiol 2020 75 03 202 208 10.1016/j.crad.2019.11.002 31858989

[21] Graves T D Condon M Loucaidou M Perry R J Reversible metronidazole-induced cerebellar toxicity in a multiple transplant recipient J Neurol Sci 2009 285(1-2):238 240 10.1016/j.jns.2009.06.011 19560788

[22] Sørensen C G Karlsson W K Amin F M Lindelof M Metronidazole-induced encephalopathy: a systematic review J Neurol 2020 267 01 1 13 10.1007/s00415-018-9147-6 30536109

[23] Yu J Fan Y Wang L Huang Y Xia J Ding L Intestinal Surgery Contributes to Acute Cerebellar Ataxia Through Gut Brain Axis Front Neurol 2019 10 995 10.3389/fneur.2019.00995 31616359 PMC6764330

